# Changes in Bioavailability of Omega-3 (DHA) through Alpha-Tocopheryl Phosphate Mixture (TPM) after Oral Administration in Rats

**DOI:** 10.3390/nu9091042

**Published:** 2017-09-20

**Authors:** Roksan Libinaki, Paul D. Gavin

**Affiliations:** Phosphagenics Limited, Unit A8, 2A Westall Road, Clayton, Melbourne, VIC 3168, Australia; pgavin@phosphagenics.com

**Keywords:** omega-3, DHA, tocopheryl phosphate mixture (TPM)

## Abstract

Benefits of Omega-3 Docosahexaenoic acid (DHA) supplements are hindered by their poor solubility and bioavailability. This study investigated the bioavailability of various formulations of Omega-3 and tocopheryl phosphate mixture (TPM), following oral administration in rats, and assessed whether TPM could improve the oral absorption of DHA. The rats were administered with a high (265.7 mg/kg) or low dose (88.6 mg/kg) of DHA. TPM was examined at 1:0.1 w/w (low TPM dose) and 1:0.5 w/w (high TPM dose). Over 24 h, the DHA plasma concentration followed a TPM dose-dependent relationship, reflected in the higher mean C_max_ values (78.39 and 91.95 μg/mL) and AUC values (1396.60 and 1560.60) for the low and high TPM, respectively. The biggest difference between the low dose DHA control (LDCont) and TPM formulations was at 4 h after supplementation, where the low and high TPM showed a mean 20% (ns) and 50% (*p* < 0.05) increase in DHA plasma concentrations versus the control formulation. After correcting for baseline endogenous DHA, the mean plasma DHA at 4 h produced by the LD-HTPM was nearly double (90%) the LDC control (*p* = 0.057). This study demonstrated that co-administering omega-3 with TPM significantly increases the bioavailability of DHA in the plasma, suggesting potential use for commercially available TPM + DHA fortified products.

## 1. Introduction

Omega-3 polyunsaturated fatty acids (PUFAs) are essential fatty acids, typically 18, 20, or 22 carbon atoms in chain length [1]. Two important dietary long chain n-3 PUFAs are docosahexaenoic acid (DHA; 22:6n-3) and eicosapentaenoic acid (EPA; 20:5n-3), which are most often found in fish oil and are also commercially available as supplements [2]. Absorption DHA and EPA directly from diet or supplements is the only practical way to increase levels of these fatty acids in the body [3].

Omega-3 supplementation, and especially DHA, is associated with improvements in cardiovascular disease, rheumatoid arthritis, and high blood pressure [4,5]. Furthermore, Omega-3 deficiency may contribute to the development of psychiatric disorders, increased inflammatory processes, poor foetal development, increased risk of cardiovascular disease, and increased risk of Alzheimer’s disease [6,7]. Despite this, Western diets are relatively low in omega-3 fatty acids [8]. Modern agriculture and farming practices have decreased the DHA omega-3 content in many foods, including meat, eggs, and fish [9,10,11]. Whereas human beings evolved on a diet balanced in omega-3 and omega-6 essential fatty acids (i.e., 1:1), the methods used to increase food production have swung the balance dramatically toward omega-6 and reduced overall omega-3 consumption.

Domesticated livestock and poultry, which are a source of omega-3 precursors, are now predominantly reared on omega-6 rich seed based diets instead of plant based diets rich in omega-3 [12,13]. In addition to this, the availability of fast and highly processed foods in the Western diet have also produced an increased consumption of saturated fats, PUFAs and other unhealthy diet options, with a concomitant decrease in consumption of oily fish [6]. Far from the optimal 1:1 ratio of omega-6/3, the current, highly processed, Western diets show a dangerous 20:1 excess in favour of omega-6 [13]. This imbalance favours a more pro-inflammatory response in the body, and is thought to contribute to the prevalence of a range of inflammatory diseases including atherosclerosis, obesity, and diabetes [13]. The relative paucity of omega-3 in the Western diet has elevated supplements to the primary contributors of DHA and EPA in the body [6].

The utilisation of the beneficial effects of omega-3 supplements in the body, are limited by the extremely low water solubility and oral bioavailability of the fatty acid [8]. Ideally, lipid-based formulations are needed to improve the bioavailability of omega-3 [14,15,16,17]. The differences in the lipid structure in which DHA and EPA are delivered may influence their bioavailability. However, most current formulations are limited by moderate solubility, increased dispersion, and poor lipid digestion [18]. Taking the supplements with high fat meals can help improve their bioavailability. However, this necessitates a high fat diet and timely intake of the supplements with the meal, which may affect compliance [19]. Emulsified fish oil preparations can also improve the digestion and enhance absorption of DHA and EPA compared with capsular supplements [20,21]. Indeed, clinical studies have demonstrated that emulsified preparations of DHA/EPA exhibits a high degree of efficient absorption compared with capsular form [20,21]. However, the emulsifiers have to be carefully considered, taking into account physical instability and oxidative protection [22].

There is a need for biocompatible and stable lipidic materials that support solubilisation during dispersion and lipid digestion. Alpha-tocopheryl phosphate mixture (TPM) is a safe [23] new lipidic material made up of two phosphorylated forms of vitamin E. TPM forms vesicles that encapsulate and solubilise poorly soluble materials [24], and has been shown to have range of positive effects when given orally [25,26,27,28,29]. Recent data have demonstrated that TPM can increase the in vitro solubility of a model lipophilic nutrient, co-enzyme Q_10_ [24]. Consequently, further work was conducted to investigate the hypothesis that TPM’s solubilizing properties may enhance the oral delivery of CoQ_10_ when compared to less solubilizing lipid formulations. TPM was first combined with medium chain triglyceride (MCT) as a formulation for CoQ_10_, and subjected to in vitro digestion experiments testing CoQ_10_ solubility as a marker for digestion. An increased amount of CoQ_10_ was solubilized in the aqueous colloidal phase throughout the in vitro digestion of the formulation (three to four-fold), in comparison to the MCT lipid formulation without TPM [30]. The relative increase in solubility produced by the TPM was the same under both fed and fasted conditions [30]. This improvement in drug solubilisation during in vitro digestion experiments was shown to translate to in vivo pharmacokinetic studies [31]. Fasted rats were administered a single oral dose of CoQ_10_ in MCT, in the presence and absence of TPM, and plasma concentrations determined over 24 h. TPM significantly increased the CMAX and AUC by approximately two-fold when compared to the MCT control formulation [31]. The increases in CoQ_10_ solubility produced by TPM during in vitro digestion were clearly shown to correlate to superior in vivo bioavailability in rats. This correlation is anticipated in situations where bioavailability is strongly linked to solubilisation in the GI tract immediately prior to absorption. These studies indicated the utility of TPM as a new lipid excipient for use in the oral delivery of poorly water soluble drugs.

The aim of this study was to investigate whether TPM could increase the oral bioavailability of a second poorly soluble nutrient, omega-3 (predominantly DHA), following a single oral administration in rats.

## 2. Materials and Methods

### 2.1. Materials

TPM was manufactured by Phosphagenics Ltd. (Melbourne, VIC, Australia). The omega-3 oil (Incromega™ DHA 500TG) was a mixture of DHA and EPA (DHA 500 mg/g; EPA 50 mg/g; CRODA, Wetherill Park, NSW, Australia). Commercially available food grade canola oil (composed of: oleic acid (~61%); linoleic acid (~21%) and α-linolenic acid (~11%)) was sourced as a vehicle for these studies. TPM formulations were prepared by dissolving the TPM (powder) into canola oil with mixing and heating (40 °C). Once cooled, the omega-3 oil was then mixed with the appropriate TPM canola oil preparation (w/w) to the required TPM/omega-3 ratio and concentration as described in the Table 1. Control formulations containing omega-3 oil (Incromega™ DHA 500TG) without TPM were diluted with canola oil to the required amount for equivalent dosing. All formulations were administered immediately after the addition of omega-3 to minimise oxidation.

### 2.2. Animals

Male Sprague Dawley rats weighing between 200 and 300 g were used for all studies. The animals were housed with two or three rats per cage and allowed to acclimatise for at least seven days before experimentation. Animals had access to food and water ad libitum throughout the treatments. Animals were administered the appropriate dose of each treatment via oral gavage and then each rat was isolated in a standard cage until the conclusion of the experiment. All animal experiments were conducted as per the approval from Monash University Animal Ethics Committee (Victoria, Australia), under approval number: SOBS/B/2008/47.

## 3. Methods

### 3.1. Study Design

Male Sprague Dawley rats were randomized into five groups (*n* = 10) to test different amounts of omega-3 in combination with TPM. Doses of omega-3 were expressed as the amount of DHA administered, which was the predominate omega-3 in the dose (10-fold higher than EPA). Two different doses of DHA were administered to rats; a low (88.6 mg/kg body weight) and high (265.7 mg/kg) dose representing a human equivalent dose (HED) range of 2.6 and 0.9 g/day. Human equivalent doses were calculated as per the FDA draft guidance document [32].

TPM was included in the oral formulations at two different DHA/TPM ratios; 1:0.1 w/w (low TPM dose) and 1:0.5 w/w (high TPM dose). The treatment groups examined using these dosing combinations are described in Table 1.

### 3.2. Administration of Formulations and Sample Collection

All lipid solution formulations were made immediately prior to administration via oral gavage. All treatment groups were dosed at the same time of day to ensure any diurnal variation in endogenous plasma concentration was equivalent between groups. The amount administered was corrected for each animal to provide the appropriate dose of DHA per body weight. On average, the volume of the oral gavage was ~1 mL. Prior to blood collection, animals were gently restrained and the tail swabbed with alcohol to disinfect the skin. A new scalpel was used for each animal to minimally nick the tail. Blood samples (250 μL) were collected in lithium heparin tubes at *t* = 0 immediately prior to dosing, and at 0.5, 1, 2, 4, 8, and 24 h post dosing. Immediately after collection, blood samples were centrifuged at 4 °C for 5 min at 8000 rpm and plasma collected. Samples were stored at −80 °C until analysis. Plasma samples were extracted and analysed for omega-3 content as per Ghasemifard et al. (2015) [33].

### 3.3. Calculation of Pharmacokinetics Parameters

Rats that completed the full treatment period with seven evaluable blood samples were included in the pharmacokinetic analysis. The pharmacokinetic results were plotted as DHA concentration (μg/mL) vs. time (h). Pharmacokinetic parameters C_max_ and T_max_ were determined for each formulation, and the truncated area under the curve (AUC_0–24_) was calculated using the trapezoidal rule. As DHA is an endogenous compound, quantifiable amounts of DHA were detected at T = 0 in rats prior to treatment administration. These values were subtracted from the plasma profiles to provide a further comparison of the relative absorption of the administered DHA. Statistical analysis was performed using an independent-samples *t*-test for comparisons assuming equal variances and a one-way analysis of variance (ANOVA) with Tukey’s multiple comparison. Statistical significance was assumed when *p* < 0.05.

## 4. Results

### DHA Levels

The plasma profiles for DHA were broadly typical for lipid based formulations, but with clear differences in exposure across the different formulations. Table 2 describes the derived pharmacokinetic parameters for DHA per treatment.

**Figure 1:**
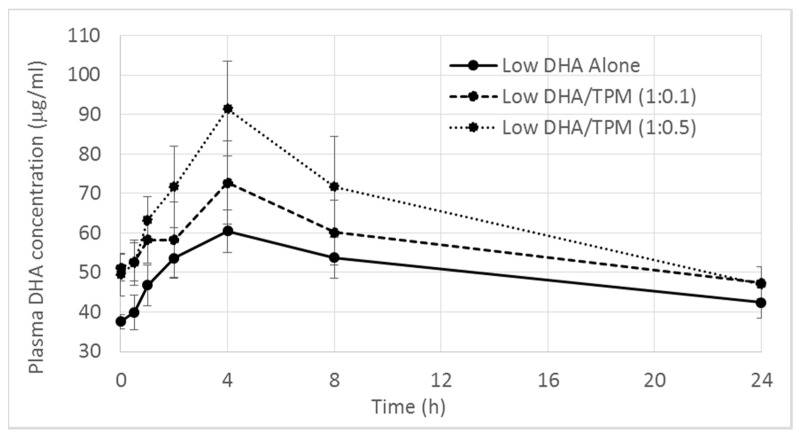
Mean DHA plasma concentrations versus time following single oral doses of low omega-3 formulations with increasing TPM (bars represent SEM).

The three low DHA treatment groups are presented first. Basal plasma concentrations of DHA averaged 40 μg/mL for the low DHA alone group and peaked at approximately 4 h following dosing then decreased to just above basal levels by 24 h post dosing (Figure 1). The low DHA alone group demonstrated a mean T_max_ of 4.56 h and a mean C_max_ of 67.20 μg/mL. The profiles for the formulations containing TPM were visibly greater in overall exposure compared to the control group. Over 24 h, the DHA plasma concentration appeared to follow a TPM dose-dependent relationship, which was reflected in the higher mean C_max_ values (78.39 and 91.95 μg/mL) and AUC values (1396.60 and 1560.60) for the low (low DHA/TPM (1:0.1)) and high TPM (low DHA/TPM (1:0.5)) respectively. The biggest difference between the low DHA alone and TPM treatment formulations was at 4 h, where the low DHA/TPM (1:0.1) and low DHA/TPM (1:0.5) groups showed a mean 20% and 50% increase in DHA plasma concentrations relative to the low DHA alone. Of these, the mean 50% increase produced by the Low DHA/TPM (1:0.5) was statistically significant (*p* < 0.05). After correcting for the baseline endogenous DHA, the mean plasma DHA concentration at 4 h produced by the low DHA/TPM (1:0.5) was nearly double (90%) the low DHA alone control, although this value fell just outside statistical significance (*p* = 0.057). There were no significant differences between the PK parameters produced by the two doses of TPM. When taken together, the improved delivery at 4 h and AUC reported for the low DHA/TPM (1:0.5) group when compared to the control, strongly supports the contention that TPM can increase the bioavailability of low doses of DHA.

**Figure 2:**
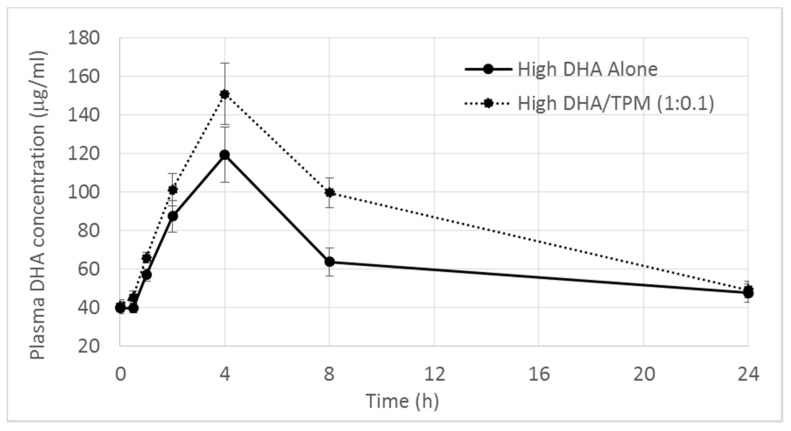
Mean omega-3/DHA plasma concentrations versus time following single oral doses of high omega-3 formulations with and without TPM (bars represent SEM).

The ability of TPM to increase DHA bioavailability replicated at higher doses of DHA. The high DHA alone group had a mean Cmax value of 124.80 mg/mL, which was roughly double that of the low DHA alone (Table 2). The Tmax values for both DHA control doses remained similar. The DHA plasma profile was again increased for the high dose treatment group by the addition of TPM to the high DHA formulation (Figure 2). The mean C_max_ values of 160.50 for the high DHA/TPM (1:0.1) group was approximately 30% higher than for the DHA control, and 40% when corrected for baseline, although these differences in C_max_ were not statistically significant. The overall systemic exposure as measured by AUC was increased by 30% with high DHA/TPM (1:0.1) treatment when compared to the high DHA alone (AUC 2078.3 μg/mL·h and 1581.2 μg/mL·h respectively). When the endogenous amount of DHA was subtracted, the increase in bioavailability produced by the high DHA/TPM (1:0.1) formulation was 80%. Both the corrected and uncorrected increases in AUC were statistically significant (*p* < 0.05).

The results for the high DHA treatment groups confirm that TPM was able to significantly increase the oral bioavailability of DHA.

Given the formulations contained relatively small amounts of EPA, the plasma samples were also analysed for EPA content. The AUC for the EPA plasma concentration profile was also significantly increased (by 40%) by the High DHA/TPM (1:0.1) formulation, when compared to the high DHA alone (*p* < 0.05; data not shown).

## 5. Discussion

Benefits related to general omega-3 supplementation are specifically attributed to DHA [34,35,36]. The DHA concentration has been shown to be particularly high in retinal and brain membrane phospholipids, and it is widely believed that DHA is involved in visual and neural function development, particularly in foetuses and young children [2]. DHA supplementation is widely believed to be beneficial in individuals with a history of heart disease, premature infants, and for supporting healthy brain development, particularly in younger children [2,4,37]. Indeed, these benefits support the choice of many manufacturers to include DHA in their products e.g., DHA-fortified dairy products. However, there is less DHA available in the average diet compared with the past due to an increase in the consumption of highly processed foods in the Western diets and decreased consumption of oily fish [12,13].

Furthermore, the beneficial properties of DHA are hindered by the low water solubility and subsequent low oral bioavailability of the fatty acid [8]. Various formulations have been employed previously to improve the oral bioavailability of DHA/omega-3 [38,39,40,41]. However, these formulations require that the supplementation takes place under specific dietary conditions (i.e., dietary restrictions), or are limited by moderate solubility, increased dispersion, and poor lipid digestion. Although others have shown, pre-emulsifying DHA in a liquid can increase DHA bioavailability by ~22-fold in humans, under fasted conditions compared with ingestion of unemulsified capsules [42], careful consideration of such emulsifiers is needed due to their physical instability and requirement for oxidative protection. 

In this study, omega-3 DHA formulated with TPM increased the bioavailability of DHA versus control treatments as measured by C_max_ and AUC in plasma vs. control. The increase in DHA plasma concentration was TPM dose-dependent in the low DHA treatment groups. Although the improved DHA bioavailability in the current study was demonstrated in fed rats, previous work undertaken in our laboratory has shown improved bioavailability of another fat soluble nutrient (coenzyme Q_10_) compared to controls in both the fed and fasted states [30,31]. It may be that TPM can replace or complement the gastric emulsification usually required for efficient oral absorption. The amount of TPM used in the current study was up to 10 times less than that examined with CoQ_10_, and no formulation work has yet been conducted to optimise the ratio of TPM to DHA. The ratio of TPM to active ingredient is important to maximise absorption. Too much TPM would dilute the DHA amongst individual TPM vesicles/micelles, increasing the number of these particles that need to be absorbed in order to deliver the intended dose. Too little TPM may lead to incomplete DHA emulsification and poorer absorption. The effect of varying TPM was evident when comparing the low DHA/TPM (1:0.1) and low DHA/TPM (1:0.5) groups. It is anticipated that formulation optimisation to identify the ideal TPM to DHA ratio could further increase oral bioavailability. Nonetheless, this study further confirms the utility of TPM as a new lipid excipient for use in the oral delivery of poorly water soluble drugs.

While increases in oral bioavailability can be produced by other pharmaceutical excipients, including phospholipids and surfactants [43], the tocopheryl phosphates possess additional characteristics that may be of benefit. In addition to a compelling safety profile [23], TPM has been shown to provide cardioprotection [25], prevent or ameliorate atherosclerosis [26,27,28], and reduce inflammation [28,29]. Given the inflammatory challenges of highly proceeded Western diets, TPM supplementation may provide additional benefits beyond its ability to increase the oral bioavailability of PUFA supplementation. 

The principles behind the production of this new formulation are applicable to other therapeutic compounds with poor solubility in aqueous solutions, and could provide a new delivery system for such hydrophobic compounds. The results presented here warrant further investigation in clinical models to determine whether TPM can produce similar increases in bioavailability in humans.

In conclusion, this study demonstrated that co-administering omega-3 with TPM significantly increases the bioavailability of DHA in the plasma. Therefore, opportunities may exist for TPM + DHA fortified products, which can improve the bioavailability and/or efficacy of omega-3. These results warrant further investigation into the health benefits and viability of adding TPM in order to improve the bioavailability of the essential fatty acid, omega-3.

## Figures and Tables

**Table 1:** Treatment formulations administered to rats. Treatments were divided into low and high doses of DHA and used in combination with two different ratios of TPM. Doses were made up in a canola vehicle and averaged 1 g total per 250 g rat.

Dose	Low Dose DHA Treatments			High Dose DHA Treatments	
	Low DHA Alone	Low DHA/TPM (1:0.1)	Low DHA/TPM (1:0.5)	High DHA Alone	High DHA/TPM (1:0.1)
**DHA (mg/kg)**	88.6	88.6	88.6	265.7	265.7
**TPM (mg/kg)**	-	8.86	44.3	-	26.57

**Table 2:** Derived pharmacokinetic parameters for orally administered DHA in canola + TPM formulations (Data are mean ± SD *n* = 7–10). Blood samples were not collected from seven rats at all scheduled time points, (one from the Low DHA Alone, three from the Low DHA/TPM (1:0.1), one from the Low DHA/TPM (1:0.5) and two from the High DHA/TPM (1:0.5) groups) and were therefore excluded from the pharmacokinetic analysis. P values are supplied versus the control formulation for each DHA dose.

Parameter	Low Dose DHA			High Dose DHA	
	Low DHA Alone (*n* = 9)	Low DHA/TPM (1:0.1) (*n* = 7)	Low DHA/TPM (1:0.5) (*n* = 9)	High DHA Alone (*n* = 10)	High DHA/TPM (1:0.1) (*n* = 8)
**T_max_ (h)**	4.56 ± 2.79	3.33 ± 0.94	4.44 ± 1.33	3.2 ± 1.03	4.25 ± 1.67
**C_max_ (µg/mL)**	67.20 ± 18.11	78.39 ± 25.21	91.95 ± 37.04	124.80 ± 37.19	160.50 ± 35.93
***p*-value vs. control**	-	*p* = 0.780	*p* = 0.186	-	*p* = 0.057
**AUC_0–24 h_ (µg/mL·h)**	1208.00 ± 281.40	1396.60 ± 411.07	1560.60 ± 638.23	1581.18 ± 372.94	2078.30 ± 319.02
***p*-value vs. control**	-	*p* = 0.730	*p* = 0.290	-	*p* = 0.007

T_max_ = the time after administration of DHA, when the maximum plasma concentration is reached. C_max_ = the peak or maximum plasma concentration of DHA.
